# Probability estimation of a Carrington-like geomagnetic storm

**DOI:** 10.1038/s41598-019-38918-8

**Published:** 2019-02-20

**Authors:** David Moriña, Isabel Serra, Pedro Puig, Álvaro Corral

**Affiliations:** 1 Barcelona Graduate School of Mathematics (BGSMath), Edifici C, Campus Bellaterra, E-08193 Barcelona, Spain; 2 Departament de Matemàtiques, Universitat Autònoma de Barcelona (UAB), E-08193 Barcelona, Spain; 3 Unit of Infections and Cancer - Information and Interventions (UNIC - I&I), Cancer Epidemiology Research Program (CERP), Catalan Institute of Oncology (ICO)-IDIBELL, L’Hospitalet de Llobregat, Barcelona, Spain; 4 Centre de Recerca Matemàtica, Edifici C, Campus Bellaterra, E-08193 Barcelona, Spain; 5 Barcelona Supercomputing Center, E-08034 Barcelona, Spain; 6 Complexity Science Hub Vienna, Josefstädter Straβe 39, 1080 Vienna, Austria

## Abstract

Intense geomagnetic storms can cause severe damage to electrical systems and communications. This work proposes a counting process with Weibull inter-occurrence times in order to estimate the probability of extreme geomagnetic events. It is found that the scale parameter of the inter-occurrence time distribution grows exponentially with the absolute value of the intensity threshold defining the storm, whereas the shape parameter keeps rather constant. The model is able to forecast the probability of occurrence of an event for a given intensity threshold; in particular, the probability of occurrence on the next decade of an extreme event of a magnitude comparable or larger than the well-known Carrington event of 1859 is explored, and estimated to be between 0.46% and 1.88% (with a 95% confidence), a much lower value than those reported in the existing literature.

## Introduction

Solar activity, through solar flares, coronal mass ejections, and solar wind, strongly influences the Earth’s magnetosphere, triggering there a range of complex phenomena such as geomagnetic storms and aurorae. These phenomena are characterized by highly intermittent and turbulent dynamics, and have also been related to a self-organized-critical state^1^. A geomagnetic storm is a disturbance in the magnetosphere quantified by changes in the Dst (disturbance-storm time) index^2,3^. This index measures the globally averaged change of the horizontal component of the Earth’s magnetic field at the magnetic equator and it is recorded once per hour^4^. During quiescent times, the Dst index varies between −20 and +20 nT (nanotesla).

**Figure 1:**
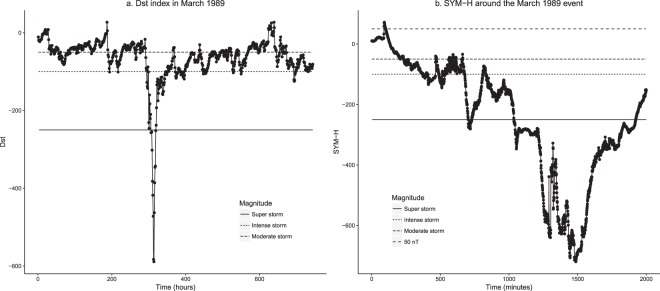
Evolution of the Dst index in March 1989 (**a**) and values of the SYM-H index for the extreme geomagnetic storm of that month (**b**). Horizontal lines mark different thresholds. Time origins are 1989-03-01 (**a**) and 1989-03-13 (**b**).

Three phases can usually be identified in a geomagnetic storm^2^. The initial phase, which is not present in all storms and is also known as storm sudden commencement, is characterized by Dst (or its one-minute component, SYM-H) increasing by 20 to 50 nT in a few minutes (but notice that not all sudden increases lead to a storm). In the main phase, whose duration is typically several hours, the Dst decreases to less than −50 nT (the more it decreases, the more intense the storm). Finally, the Dst returns to its quiescent-time range in the recovery phase, a process that can be quite erratic and may last from hours to days. In general, two consecutive storms with less than 48 hours of difference between them are considered to be the same^5^. Figure 1(a) shows the time evolution of the Dst index in March 1989, including the most intense event on record; the storm sudden commencement at the beginning of the event can be noticed on Fig. 1(b), showing the SYM-H evolution.

The minimum Dst value reached during the main phase (always negative) will give the intensity of the storm (also referred to as magnitude). This allows one to distinguish three categories of geomagnetic storms: super-storms (under −250 nT), intense (under −100 nT) and moderate (under −50 nT). The introduction of an intensity threshold in the original Dst time series leads to the fact that the storms defined in this way constitute a point process where the occurrence times of the events are the times of threshold crossing from above. The potential effects of geomagnetic storms on human life are discussed in the next section.

The Dst index has been traditionally modelled by means of its physical properties^3,6^, although some work has also focused on exploring its statistical properties^7^. As far as we know, all efforts in statistical modelling have been based on the assumption that the occurrence of a geomagnetic storm follows an homogeneous Poisson counting process (see for instance ref.^3^). In general, a counting process *N*(*t*) arises when dealing with the number of events occurring over time, and its rate *λ*(*t*) is a stochastic process defined by

1 $$\lambda (t)=\frac{1}{dt}P(N(t+dt)-N(t)=1|{\rm{past}}),$$

where *P* denotes probability (with the vertical bar for conditional probability) and *N*(*t*) counts the number of events from the time origin up to time *t*. The simplest case arises for a homogeneous Poisson (counting) process, in which the rate *λ*(*t*) = *λ* is constant, and this leads to the fact that the times between consecutive events, known as inter-occurrence times, are independent and exponentially distributed^8^. For a non-homogeneous Poisson process, *λ*(*t*) is still independent on the past events, but evolves in time in a predetermined way. This independence constitutes the well-known lack-of-memory property of the Poisson process.

As shown in this paper, the homogeneous-Poisson assumption for the occurrence of geomagnetic storms is not supported by statistical testing. In consequence, we propose a model based on a Weibull counting process, a generalization that contains the homogeneous Poisson process as a particular case (an analogous model was introduced and applied to demographic data in ref.^9^). By means of the Weibull inter-occurrence times, the model allows us to compute the probability of threshold surpassing of future values of the Dst index over different time windows, and can be extrapolated to the most extreme geomagnetic storms thanks to the stability of the shape parameter and the exponential dependence of the Weibull scale parameter with the intensity threshold.

## Effects of Geomagnetic Storms

Geomagnetic storms of remarkable intensity may have a potential effect on many aspects of human life, including health effects due to radiation hazards, disruption of electrical systems like communications appliances, navigation systems, satellites… The biological mechanisms altered by geomagnetic storms are explored in ref.^10^, and geomagnetic activity has been recently related to several human conditions as hypertension^11^, multiple sclerosis^12–14^ or cancer^15^. However, it is still unclear to what extend and under what threshold these health effects appear or the risk increases. Regarding communications, in the period under study the most intense event happened on March 1989, reaching a peak Dst under −600 nT, Fig. 1, which caused the collapse of the Hydro-Québec power grid, and resulted in the loss of electrical power to six million people^16^. Another event on July 15-16, 2000 with a peak Dst under −300 nT did not cause any terrestrial damage. Therefore, it seems reasonable to infer that the threshold to expect immediate observable consequences might be between these two values. In fact, some authors have speculated about the potential effects of an extreme geomagnetic event on today’s society^17,18^.

The Carrington event is the largest known example of geomagnetic storm, occurred by the end of August and early September 1859 and is associated to a minimum Dst under −850 nT^19^. Richard C. Carrington was observing sunspots on the solar disk and saw a large solar flare with optical brightness equaling that of the background sun, lasting several minutes and due to the destabilization of a large region of the sun causing an extremely fast coronal mass ejection towards Earth. These observations allowed to notice a delay of about 18 hours between the flare and the storm, explained in the recent literature in terms of propagation of the solar wind^20^. It is known that this geomagnetic storm had an effect on the telegraphic network, as several stations reported issues during these days, in different locations around the globe. Some stations were unusable for some hours, but there is no evidence that this interruption on the telegraph service had any impact on the economic activity^18^.

## Data and Storm Definition

To analyse the process of temporal occurrence of geomagnetic storms we use the Dst index, recorded hourly from 1957-01-01 to 2017-12-31 and available from the World Data Center for Geomagnetism in Kyoto^21^. When the Dst signal crosses a fixed negative threshold from above this defines the occurrence time or starting time of a geomagnetic storm with an intensity limited by the threshold; the storm ends when the signal gets over the threshold again. In this paper we consider different thresholds, ranging between −50 nT to −400 nT in steps of −10 nT, although as discussed in the next section only storms under −150 nT are used to build the model that allows forecasting.

The time between two consecutive storms (for a given threshold in Dst) is just the difference of their occurrence times, which defines the inter-occurrence time (measured in days, unless otherwise stated, and called waiting time in refs^22–25^). As the minimum inter-occurrence time to consider two storms as different (and not part of the same storm) is usually taken to be two days^5^, inter-occurrence times below this value are discarded in our procedure. This prevents overcounting arising from small spurious events that appear when the Dst signal remains around the threshold accidentally. As a sensitivity measure, we have explored alternative storms definitions, changing the inter-occurrence time threshold from two days to other (fixed) values, or defining a storm only if its duration is larger than a given fixed period (i.e., an event is considered as such only if the index remains below the threshold for at least 6 hours, for example). These different approaches lead to very similar final results.

## The Model

**Figure 2:**
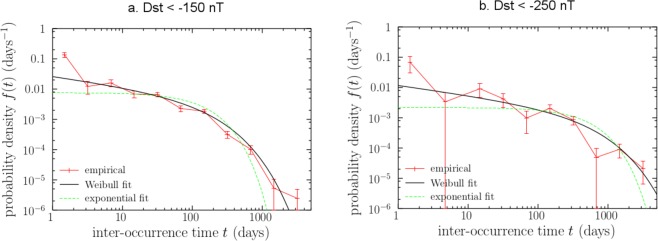
Empirical and fitted Weibull distributions for the time between consecutive geomagnetic storms, defined using two different thresholds, *T* = −150 nT (**a**) and *T* = −250 nT (**b**). The distributions are represented in terms of their probability densities, *f*(*t*). The empirical ones have been estimated with the so-called “log-binning” method^56^. Exponential fits, shown for comparison, underestimate the probability of both short and long times. Times below two days (disregarded in our approach and also in these fits) are grossly underestimated by any of the models. Weibull fits use the parameters coming from Eq. (2). Plotted using Gnuplot (version 3.7), see ref.^57^.

As already mentioned, the time of occurrence of geomagnetic storms has been traditionally analysed in the literature as a homogeneous Poisson process^3^. However, the homogeneous-Poisson-process hypothesis can be tested by checking if inter-occurrence times are exponential, and according to Fig. 2, this distribution seems to be far from doing the fitting optimally. Instead, inter-occurrence times seem to be well fitted by Weibull distributions (see again Fig. 2). In terms of the complementary cumulative distribution function, *S*(*t*) = *P*(*X* > *t*), the Weibull distribution takes the form

$$S(t)={e}^{-{(t/\tau )}^{\gamma }},$$

where *X* is the random variable representing inter-occurrence time, *t* a concrete value, *γ* the shape parameter and *τ* the scale parameter. The Weibull probability density is obtained as *f*(*t*) = −*dS*(*t*)/*dt* and the rate, or hazard rate, as *λ*(*t*) = *f*(*t*)/*S*(*t*). Our choice of the Weibull distribution is based on purely empirical grounds, as a common generalization of the homogeneous Poisson process, which is recovered as a particular case just setting *γ* = 1. Nevertheless, in the Discussion Section we speculate about the origin of the Weibull distribution (a derivation of it in the context of catastrophic failure of materials is given in ref.^26^).

For instance, for super-storms (Dst < −250 nT), a Weibull distribution with shape parameter *γ* = 0.63 and scale parameter *τ* = 329 days fits well the inter-occurrence times; this can be verified by means of a Lilliefors-corrected Kolmogorov-Smirnov test (p-value = 0.11) conducted using the R package *KScorrect*^27^. By means of the same technique, it can also be seen that an exponential distribution does not fit the data properly (p-value < 0.0001, which rules out the homogeneous Poisson process). As the shape parameter *γ* is below one, this Weibull distribution has a decreasing failure rate (DFR) or decreasing hazard rate, and therefore the associated count distribution (number of storms within this threshold in a period of time) should be overdispersed^28^, confirmed by a dispersion index $d=\frac{{\sigma }^{2}}{\mu }=2.10$ (i.e., above one), where *σ*^2^ and *μ* are, respectively, the sample variance and the sample mean of the number of super-storms in one year during the study period.

**Figure 3:**
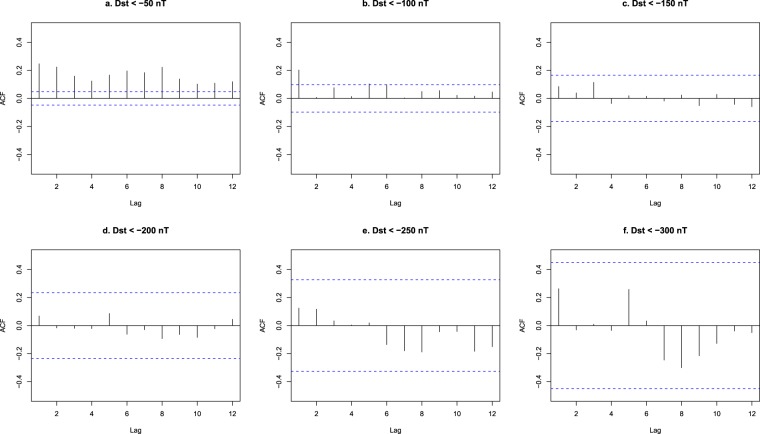
Autocorrelation function of the inter-occurrence times for moderate (**a**), intense (**b**), Dst < −150 nT (**c**), Dst < −200 nT (**d**), super-storms (**e**) and Dst < −300 nT (**f**). The horizontal axis (lag) counts storm separation in terms of number of storms. Horizontal lines denote the limits of 95% confidence intervals.

Regarding correlations in the inter-occurrence times, geomagnetic storms defined by intensity thresholds equal or below −150 nT show no significant temporal autocorrelations, as shown in Fig. 3 (the confidence intervals showed in Fig. 3 are computed taking into account that the autocorrelation coefficient at lag *k*, ${\hat{\rho }}_{k}$, under the null hypothesis that the observations are independent and identically distributed, is asymptotically normal with variance $VAR({\hat{\rho }}_{k}) \sim \frac{1}{N}$, where *N* is the number of observations, see ref.^29^); nevertheless, significant correlations are found for weaker events. This prevents the application of some statistical techniques to these weaker storms; therefore, to avoid temporal correlations, in what follows we focus on geomagnetic storms defined by a Dst threshold equal to or below −150 nT. These storms are, in fact, the ones that may have a deeper impact on human activity, as discussed in a previous section. However, it is important to remark that it is not that there are no correlations between the inter-occurrence times of the more intense storms, but that these correlations cannot be established as statistically significant.

### Forecasting

**Figure 4:**
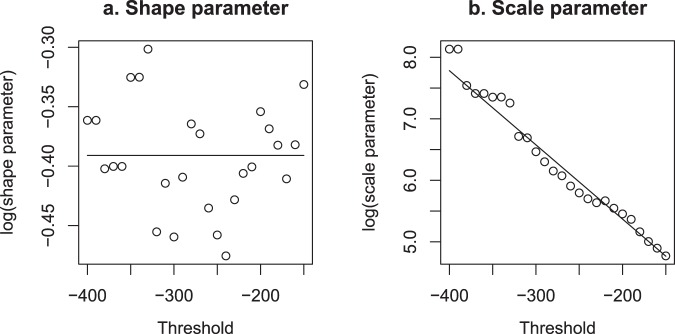
Relationship between Dst threshold (in nT) and Weibull shape (**a**) and scale (**b**) parameters, in log-scale, with scale parameter in days. Intensity thresholds range from −400 nT to −150 nT. The points correspond to maximum-likelihood estimates of the shape and scale parameters for fixed threshold values. The lines are the result of a Weibull regression model which is fitted directly to the whole data set of inter-occurrence times and thresholds (not to the shape and scale parameters).

The proposed model can be used to estimate the probability of an event below a certain threshold in Dst to occur in a period of time, based on studying the relationship between the threshold and the Weibull shape and scale parameters. This relationship can be even used to forecast extreme events with a threshold outside the observed range. It can be seen (Fig. 4) that the shape parameter does not depend on the threshold while a linear relationship seems to appear for the logarithm of the scale parameter and the threshold.

Indeed, several models were fitted to the scale parameter using the threshold in Dst as explanatory variable, and the one that showed a better fit was the linear regression model

2 $$\mathrm{log}(\tau )={\beta }_{0}+{\beta }_{1}\cdot T.$$

In consequence, the inter-occurrence times were finally fitted using a Weibull regression model (see ref.^30^) where the scale parameter *τ* changes with the threshold according to Eq. 2 and the shape parameter *γ* is constant (independent of the threshold); in other words, we fit the threshold-dependent distribution

3 $$S(t;T)=\exp [\,-\,{(t/{e}^{{\beta }_{0}+{\beta }_{1}T})}^{\gamma }],$$

using R *survival* package (ref.^31^). The inter-occurrece times analyzed were those arising from the thresholds *T* = −150, −160, −170, … up to −400 nT. Both scale and shape parameters can be recovered from the Weibull regression model ($\tau ={e}^{{\beta }_{0}+{\beta }_{1}\cdot T}$, in units of days). The estimates are $\mathrm{log}(\hat{\gamma })=-\,0.39$ (*SE* = 0.023), ${\hat{\beta }}_{0}=2.96$ (*SE* = 0.17) and ${\hat{\beta }}_{1}=-\,0.0121$ (*SE* = 0.0008, units of nT^−1^).

Regression models based on other distributions might be appropriate according to the behavior described in Fig. 2, such as a gamma regression model, which is implicit in previous works on earthquakes and solar flares (see refs^32–34^. and in concrete Eq. 5 of ref.^35^ and Fig. 13 of ref.^36^), although not recognized there as regression models and not fitted as such. In order to justify our final choice of the Weibull regression model, the goodness of fit of the Weibull and the gamma distributions for fixed thresholds have been compared by means of the Akaike Information Criterion (AIC), showing that the fit of the Weibull model is better regardless of the threshold. The details can be found in the supplementary material (Table S1), comparing also with the standard exponential distribution, which showed the worst fit.

### Forecasting another Carrington event

Some efforts have been recently devoted to estimate the probability of another Carrington event in the next 10 years^3^. According to our results, the estimated shape parameter of the corresponding Weibull distribution is $\hat{\gamma }={e}^{-0.39}=0.68$, and Eq. 2 with threshold *T* = −850 nT leads to the scale parameter, $\hat{\tau }=563882$ days (1544 years). Knowing that the original Carrington event happened in 1859, about 58000 days ago, one can compute the probability of having a Carrington or more intense event during the next decade (2018–2027) conditioned to the fact that no event like this has happened since 1859,

4 $$P(X\,\le \,{t}_{C}\,+\,{t}_{d}|X\,\ge \,{t}_{C})=\frac{S({t}_{C})\,-\,S({t}_{C}\,+\,{t}_{d})}{S({t}_{C})}=1-\exp [{(\frac{{t}_{C}}{\tau })}^{\gamma }\,-\,{(\frac{{t}_{C}+{t}_{d}}{\tau })}^{\gamma }]=0.0092,$$

with *t*_*C*_ = 58000 days and *t*_*d*_ = 3652 days (10 years), and so, the probability is 0.92%. Note that the value reported in ref.^3^ was about 12%, in sharp contrast with our result. This important difference can be explained in part by the more flexibility of the Weibull model and in part by the fact that the tail of the Dst distribution seems to be better fitted by an exponential distribution than by a power law (p-value  = 0.1 and p-value < 0.0001 respectively, but other distributions have been proposed^37^), according to the methodology described in ref.^38^ and implemented in the R package *poweRlaw*^39^. Note that the exponential behavior of the tail of the distribution is directly related to Eq. 2.

In addition to the probability point estimate, an approximation of its standard error can be obtained by bootstrapping inter-occurrence times and thresholds and fitting the described Weibull regression model for each bootstrapped data set. Using this method the estimated standard error of the resulting probability is *SE* = 0.0037, and a 95% confidence interval can be built by computing percentiles 2.5% and 97.5%, leading to the interval (0.46%,1.87%). Taking into account that the probability in Eq. 4 is a function of *γ* and *τ*, with the latter depending on *β*_0_ and *β*_1_, its standard error could also have been estimated by using the *delta method*, leading to a very similar result of *SE* = 0.0031. The R package *car*^40^ was used with this purpose, and the R code for both methods is available as supplementary material. In the latter case, however, for the construction of the corresponding confidence interval asymptotic normality needs to be assumed but the distribution of the probabilities given by Eq. 4 obtained by bootstrapping is not symmetrical, so the bootstrap confidence interval is preferred.

The Carrington event was first estimated to have a Dst of about −1760 nT^41^. If we assume this threshold, the corresponding Weibull distribution would have shape and scale parameters 0.68 and 3.18 × 10^10^ days (8.7 × 10^7^ years) respectively, so the probability of having such an event within the next decade would be 0.0005%, with a 95% confidence interval (9.11 × 10^−5^%; 0.0033%). A probability of 1.5% was reported in ref.^3^, again much higher than the values in our interval.

Using the expectation of the underlying Weibull counting process *N*(*t*), the expected number of storms with intensity below a specific threshold can be calculated for any time period *t*, and is given by the expression^9^

5 $${\bf{E}}(N(t))={\sum }_{n=1}^{\infty }{\sum }_{j=n}^{\infty }\frac{n{(-\mathrm{1)}}^{j+n}{(t/\tau )}^{\gamma j}{\alpha }_{j}^{n}}{{\rm{\Gamma }}(\gamma j+\mathrm{1)}},$$

where ${\alpha }_{j}^{0}=\frac{{\rm{\Gamma }}(\gamma j+1)}{{\rm{\Gamma }}(j+1)},j=0,1,\ldots$ and ${\alpha }_{j}^{n+1}={\sum }_{m=n}^{j-1}{\alpha }_{m}^{n}\frac{{\rm{\Gamma }}(\gamma j-\gamma m+1)}{{\rm{\Gamma }}(j-m+1)},n=0,1,\ldots$ and *j* = *n* + 1, *n* + 2,…. The formula is exact when a below-threshold event (which is not counted) occurred at the beginning of the time period.

For instance, refs^17,42^ reported a table including the estimated frequencies for geomagnetic storms with different thresholds. Our method yields substantially different results, as shown in Table 1. To obtain an estimation within the same order of magnitude, one could also use the asymptotic approximation ${\bf{E}}(N(t))=\frac{t}{m}$, where *m* is the average inter-occurrence time which can be calculated using the corresponding Weibull expectation, given by $\tau {\rm{\Gamma }}(1+\frac{1}{\gamma })$.

**Table 1:** Estimated frequencies for geomagnetic storms of different thresholds, based on the exact expression (Eq. 5), calculated for the indicated time periods.

Threshold (nT)	Frequency (Eq. 5)
−100	4.95 per 1 year
−200	1.78 per 1 year
−400	1.63 per 10 years
−800	1.37 per 1,000 years
−1600	0.19 per 1,000,000 years

## Discussion

Several authors have proposed models in order to forecast the occurrence of geomagnetic storms of certain magnitude^3,7,43,44^, most of them based on physical properties of the Dst index, like in refs^6,45^. To date, the probabilistic approaches to forecast these events have been based, to our knowledge, on the assumption that the number of storms in a given period of time follows an homogeneous Poisson process^3,5^. This assumption might be violated, especially for intense and super-storms, as we have verified following the Lilliefors-corrected Kolmogorov-Smirnov test^27^, as also displayed visually in Fig. 2.

We have introduced a Weibull counting process which includes the homogeneous Poisson process as a particular case. The model is defined, for any intensity threshold, by independent Weibull inter-occurrence times, with a constant (independent on the threshold) and smaller-than-one shape parameter, and a scale parameter depending exponentially on the intensity threshold, Eq. 2. The estimated probability of occurrence of a Carrington or more extreme event (Dst < −850 nT) in the next decade using the proposed model (~0.92%) is much lower than the values estimated in previous works using a different probabilistic approach (ref.^3^ reports a probability around 12%) and the estimated probabilities are even more different for higher thresholds. The reason of this strong discrepancy is probably and in part due to the fact that the extrapolation to severe geomagnetic storms of a power-law^46^ probability distribution of intensities is problematic^47^. This deviation from the power-law distribution was pointed out in ref.^3^ and explored in refs^37,42^ by fitting a gamma and a lognormal distribution to the storm maximum Dst, respectively.

In addition, our Weibull regression model is flexible enough to include other covariates, in addition to the Dst thresholds. According to ref.^22^. the geomagnetic storm inter-occurrence-time distribution is strongly related to solar cycle, so a similar model based on Eq. 2 including an extra term to account for the sunspot number was fitted, leading again to a better performance of the Weibull regression model compared to gamma and exponential alternatives and a very similar shape parameter estimate of $\hat{\gamma }=0.72$ (*SE* = 0.023). This will be analyzed in depth in future research.

An interesting property of the Weibull distribution is that the hazard rate is just a power-law function, *λ*(*t*) ∝ *t*^*γ*−1^; this is invariant under change of scale, which is physically suggestive, and the Weibull distribution is the only one having this property^48^ (note that power-law distributions should also verify this property with *γ* = 0, but they are not defined for all *t* > 0). In our study, the estimated Weibull model yields a shape parameter *γ* below one, leading therefore to a decreasing hazard rate. This means that the longer since the last super-storm happened, the less the chance to happen another one in the next unit of time. Thus, the estimated probability of occurrence of a Carrington event in the first decade after 1859 is 3.33%, which is 3.6 times greater than the estimated probability for the current decade. This property in which hazard decreases over time is not only counter-intuitive but fundamental for hazard assessment, and seems to be present in the occurrence of many geological and astronomical extreme events^23,24,35,49–51^. The origin can be attributed to long-range correlations in the Dst time series, as several authors^50,52^ have argued that in such a case the inter-occurrence time statistics is of the decreasing-hazard-rate type. Note that, although the few number of storms available for too extreme Dst thresholds precludes that the correlations between the inter-occurrence time are significant, this does not rule out their existence.

Our findings also indicate some other sort of self-similarity in the temporal occurrence of geomagnetic storms (in addition to the hazard rate function becoming a power law, which is the hallmark of scale invariance for univariate functions). The fact that the shape parameter remains stable under changes in the threshold setting the severity of the storms implies that the temporal occurrence of storms follows the same dynamics for any of the analyzed threshold intensities (except for a trivial change of scale set by the scale parameter *τ*). It is noteworthy that many natural hazards share this property of self-similarity^53^, which is not linked to the particular model of the inter-occurrence times (i.e., not linked to the Weibull assumption). In the case of point events this has been observed for earthquakes^35,36^ and forest fires^54^, whereas for time series (the case under consideration here) this behavior is present in rainfall events^49^, annual climate records (temperature, rives floods, etc.^50^), and (of particular interest for its relation with geomagnetic storms) solar flares^23,24^. Further, the fact that the scale parameter *τ* depends exponentially on the threshold *T* implies that the waiting time can be rescaled as $t/{e}^{{\beta }_{1}T}$, see Eq. 3. Defining a new variable as *E* ∝ *e*^*α*|*T*|^, the same rescaling can be written as $t/{E}^{|{\beta }_{1}|/\alpha }$, which implies that Eq. 3 can be rewritten in the form of a time-energy scaling law, as found previously for earthquakes and other natural hazards^36^.

Although there are physical limitations to the possible scale-invariance of geomagnetic storms (due to theoretical upper limits to their size based on the saturation properties of the magnetospheric ring current and polar-cap conductivity, these limitations should affect the distribution of storm size, but not necessarily the temporal properties of the storms, given by Eq. (3). In other words, this equation gives scale-invariant properties no matter the size distribution of the storms. Note that time-size scale invariance without power-law distributed sizes has been claimed in other systems in geoscience^54^.

Regarding the effects of an extreme geomagnetic storm like the Carrington event, it seems reasonable to think that these would have a deeper impact nowadays than in 1859. For instance, there were no radio communications in 1859 and a global blackout of these and any communications involving ionospheric effects can be expected due to a severe geomagnetic storm. Power distribution networks would probably be the most sensitive equipment as major communication infrastructure is based on optical fibres links, which are much less sensitive to perturbations than the 1859 network. In early November 2003 an important perturbation happened in space (known as the Halloween event), although associated with a higher Dst than Carrington event’s^55^. These storms interfered with satellite communications (the Japanese ADEOS-2 was partly damaged) and produced a brief power outage in Sweden. This event and its consequences are described in ref.^17^.

## Supplementary information

SUPPLEMENTARY MATERIAL Accompanying the manuscript: Probability estimation of a Carrington-like geomagnetic storm

## Data Availability

All data generated or analysed during this study are included in this published article (and its Supplementary Information files).
