# Supplementary material for: Probability estimation of a Carrington-like geomagnetic storm
Source: Sci Rep. 2019 Feb 20;9:2393. doi: 10.1038/s41598-019-38918-8 (PMC6382914; doi:10.1038/s41598-019-38918-8)
Supplement: Supplementary file 1 — SUPPLEMENTARY MATERIAL Accompanying the manuscript: Probability estimation of a Carrington-like geomagnetic storm [file 41598_2019_38918_MOESM1_ESM.pdf]

SUPPLEMENTARY MATERIAL

Accompanying the manuscript:

Probability estimation of a Carrington-like geomagnetic storm

David Moriña[1,2,3*], Isabel Serra[1,2,4], Pedro Puig[1,2] and Álvaro Corral[1,2,4,5]

[1]Barcelona Graduate School of Mathematics, Edifici C, Campus Bellaterra, E-08193 Barcelona, Spain

[2]Departament de Matemàtiques, Universitat Autònoma de Barcelona, E-08193 Barcelona, Spain

[3]Unit of Infections and Cancer (UNIC - I&I), Cancer Epidemiology Research Program (CERP), Catalan Institute of Oncology (ICO)-IDIBELL, L'Hospitalet de Llobregat, Barcelona, Spain

[4]Centre de Recerca Matemàtica, Edifici C, Campus Bellaterra, E-08193 Barcelona, Spain

[5]Complexity Science Hub Vienna, Josefstädter Straβe 39, 1080 Vienna, Austria

*david.morina@uab.cat

1. Bootstrap

Bootstrap is a simulation method based on taking several random resamples with replacement from the original data set (in our case we have used 100,000 different resamples of the same size). Among other uses, this technique allows inferring numerical properties of a parameter (as its variance or quantiles), so a (1-α)% confidence interval can be constructed by considering the percentiles $\frac{\alpha}{2}$% and (1-$\frac{\alpha}{2}$)% of the obtained distribution of the parameter over the generated data sets.

2. Delta method

The delta method is useful to estimate the variance of a non-linear function of an estimator. It is based on the expansion of a function of a random variable about its mean, usually with a one-step multivariate Taylor approximation, and computing its variance. For example, if G is a differentiable real valued function of a k-dimension random vector X = (x_1, …, x_k) with mean vector $\mu = (\mu_1, \dots, \mu_k)$ and known variance-covariance matrix Σ, the first order Taylor expansion of $G(X)$ is

$$G(X) = G(\mu) + \sum_{i=1}^{k} \frac{\partial G}{\partial x_i}(\mu)(x_i - \mu_i), \tag{S1}$$

so the variance of $G(X)$ can be obtained as,

$$VAR(G(X)) \sim \nabla G(\mu) \cdot \Sigma \cdot \nabla^t G(\mu), \tag{S2}$$

where $\nabla G(\mu) = (\frac{\partial G}{\partial x_1}(\mu), \dots, \frac{\partial G}{\partial x_k}(\mu))$ and the dot indicates matrix product.

In R the delta method is implemented with function *deltaMethod* from the package *car*[1].

3. Weibull counting process

A counting process arises when dealing with the number of events occurring over time, being the Poisson counting process the simplest and most popular example, assuming that the times between events (inter-occurrence times) are independent and identically exponential distributed. Many other counting processes have been proposed in the literature although in most cases the close relationship between the distribution of the number of occurrences and the distribution of inter-occurrence times has not been explored. A counting process with Weibull inter-occurrence times is described in detail in Ref.[2]. The Poisson counting processes is a particular case of the Weibull counting process, which can handle situations with overdispersion and underdispersion in the number of occurrences. The event rate at time *t* is given by the *failure rate* or *hazard rate function*, defined by

$$\lambda(t) = \frac{f(t)}{S(t)},$$

where $f(t)$ is the density function and $S(t)$ is the complementary cumulative probability function. In the Weibull case $f(t) = \frac{\gamma}{\tau}\left(\frac{t}{\tau}\right)^{\gamma-1} e^{-\left(\frac{t}{\tau}\right)^{\gamma}}$, $\gamma, \tau \in \mathbb{R}^+$ and $S(t) = e^{-\left(\frac{t}{\tau}\right)^{\gamma}}$, where γ is the shape parameter and τ is the scale parameter. As shown in Ref.[3], if the hazard function is monotonic and increasing (increasing hazard rate or failure rate, IHR or IFR), the associated

number of occurrences is underdispersed, while it is overdispersed if the inter-occurrence times hazard function is monotonic and decreasing (DHR of DFR). In the Weibull case, this is related to the shape parameter as values of γ below 1 lead to DFR and values of γ over 1 lead to IFR. The case $\gamma = 1$ leads to the exponential distribution and the corresponding Poisson process.

Table S1 shows that the Weibull distribution is reasonable to fit the inter-occurrence times for storms with maximum -Dst over 150nT (intense storms), improving the goodness of fit of the exponential and gamma distributions in all cases.

Threshold (nT)	Distribution	AIC	N	p-value
**-150**	**Weibull**	1667.46	141	0.0376
	**Gamma**	1679.09		0.001
	**Exponential**	1701.85		< 0.0001
**-200**	**Weibull**	925.38	70	0.0756
	**Gamma**	943.6		0.007
	**Exponential**	942.75		< 0.0001
**-250**	**Weibull**	503.79	36	0.1214
	**Gamma**	511.69		0.0188
	**Exponential**	519.09		< 0.0001
**-300**	**Weibull**	292.57	19	0.9654
	**Gamma**	293.2		0.7678
	**Exponential**	298.78		0.032

Table S1. Goodness of fit of Weibull, gamma and exponential distributions to inter-occurrence times for several thresholds. AIC is Akaike information criterion. N is the number of events for each threshold. p-values from Lilliefors-corrected Kolmogorov-Smirnov goodness of fit test. The preferred model is that with minimum AIC.

Let X_n be the time from the measurement origin at which the *n*-th event occurs. Let $N(t)$ denote the number of events that have occurred up until time *t*. The relationship between $N(t)$ and X_n can be stated by saying that the amount of time at which the *n*-th event occurred from the time origin is less than or equal to *t* if and only if the number of events that have occurred by time *t* is greater than or equal to *n*. From this relationship, the probability function of the number of occurrences can be build as

$$P_n(t) = P(N(t) = n) = P(N(t) \geq n) - P(N(t) \geq n + 1) = P(X_n \leq t) - P(X_{n+1} \leq t). \quad \text{(S3)}$$

If we let the cumulative density function of X_n be $F_n(t)$, then $P_n(t) = P(N(t) = n) = F_n(t) - F_{n+1}(t)$. In the case where the measurement time origin (and thus the counting process) coincides with the occurrence of an event, then $F_n(t)$, is simply the *n*-fold convolution of the common interarrival time distribution which may or may not have a closed-form solution. In the Weibull case, the convolutions of the form $\int_0^t F_n(t-s)f(s)ds$ do not have a closed form, so Ref.[2] proposes a Taylor series expansion of the Weibull density

to evaluate numerically these integrals. The Weibull counting process is implemented in the R package *Countr*[4].

4. Autocorrelation function (ACF) in the time series context

In the time series context, given a series X_k with mean $\mu_k = E[X_k]$ and autocovariance function $\gamma_X(k+h,k) = Cov(X_{k+h}, X_k) = E[(X_{k+h} - \mu_{k+h})(X_k - \mu_k)]$, its autocorrelation function (ACF) is defined as

$$\rho_h = \rho_X(h) = \frac{\gamma_X(h,0)}{\gamma_X(0,0)} = Corr(X_{k+h}, X_k)$$

In our case, X_k is the k -th inter-occurrence time, with k labelling successive inter-occurrence times, $k = 0,1,2,\ldots$, and h is refered to as the lag.

5. R code to reproduce the analyses

```
library(Countr)
library(fitdistrplus)
library(KScorrect)
library(ADGofTest)
library(ggplot2)
library(car)
library(poweRlaw)
library(reshape2)
library(data.table)

load("Scientific Reports/Supplementary Material/dstDEF.RData")
load("Scientific Reports/Supplementary Material/diff.timesDEF.RData")
load("Scientific Reports/Supplementary Material/Quebec.RData")
load("Scientific Reports/Supplementary Material/sunspots.RData")

bi.test <- function(data)
{
  h <- vector()
  for (i in 3:(dim(data)[1]-2))
  {
    xpre  <- difftime(data$CDATE[i], data$CDATE[i-1], units="days")
    xpost <- difftime(data$CDATE[i+1], data$CDATE[i], units="days")
    if (xpre <= xpost)
    {
      x <- xpre
      y <- difftime(data$CDATE[i-1], data$CDATE[i-2], units="days")
    }else{
      x <- xpost
      y <- difftime(data$CDATE[i+2], data$CDATE[i+1], units="days")
    }
    h[i] <- as.numeric(x)/as.numeric((x+y/2))
  }
  pval <- ad.test(h, punif)$p.value
  return(list(h=h, pvalue=pval))
}

# Multiple plot function (used to generate figure 1)
#
# ggplot objects can be passed in ..., or to plotlist (as a list of ggplot objects)
# - cols:   Number of columns in layout
# - layout: A matrix specifying the layout. If present, 'cols' is ignored.
#
# If the layout is something like matrix(c(1,2,3,3), nrow=2, byrow=TRUE),
# then plot 1 will go in the upper left, 2 will go in the upper right, and
# 3 will go all the way across the bottom.
#
multiplot <- function(..., plotlist=NULL, file, cols=1, layout=NULL) {
  library(grid)
```

```
  # Make a list from the ... arguments and plotlist
  plots <- c(list(...), plotlist)

  numPlots = length(plots)

  # If layout is NULL, then use 'cols' to determine layout
  if (is.null(layout)) {
    # Make the panel
    # ncol: Number of columns of plots
    # nrow: Number of rows needed, calculated from # of cols
    layout <- matrix(seq(1, cols * ceiling(numPlots/cols)),
                     ncol = cols, nrow = ceiling(numPlots/cols))
  }

  if (numPlots==1) {
    print(plots[[1]])

  } else {
    # Set up the page
    grid.newpage()
    pushViewport(viewport(layout = grid.layout(nrow(layout), ncol(layout))))

    # Make each plot, in the correct location
    for (i in 1:numPlots) {
      # Get the i,j matrix positions of the regions that contain this subplot
      matchidx <- as.data.frame(which(layout == i, arr.ind = TRUE))

      print(plots[[i]], vp = viewport(layout.pos.row = matchidx$row,
                                      layout.pos.col = matchidx$col))
    }
  }
}

### Test the Poisson assumption
bi.test(dst[dst$STORM25==1, ])

### Test if inter-occurrence times are Weibull or exponential for super-storms
LcKS(na.omit(diff.times$diff25), cdf="pweibull")$p.value
LcKS(na.omit(diff.times$diff25), cdf="pexp")$p.value

### Test if Dst tails follow a power law / exponential distribution
dst_tail <- dst[dst$DST<0, ]
m_pl <- conpl$new(-1*dst_tail$DST) ### power law
est <- estimate_xmin(m_pl)
m_pl$setXmin(est)
bootstrap_p(m_pl, threads = 4)$p
m_pl <- conexp$new(-1*dst_tail$DST) ### exponential
est <- estimate_xmin(m_pl)
m_pl$setXmin(est)
bootstrap_p(m_pl, threads = 4)$p

### Anual number of storms dispersion index (Dst < -250nT)
data <- dst[dst$STORM25==1, c(1, 2, 27)]
data$YEAR <- as.numeric(substr(data$CDATE,1,4))
for (i in 2:dim(data)[1])
{
  if (as.numeric(difftime(data$CDATE[i], data$CDATE[i-1])) < 2) data$STORM25[i] <- 0
}
eval(parse(text=paste0("df.YEAR <- aggregate(data$STORM25, by=list(data$YEAR), FUN=sum)")))
while(dim(df.YEAR)[1]!=(df.YEAR$Group.1[length(df.YEAR$Group.1)]-df.YEAR$Group.1[1]+1))
{
  for (i in 2:dim(df.YEAR)[1])
  {
    if ((df.YEAR$Group.1[i] != df.YEAR$Group.1[i-1]+1))
    {
      df.YEAR <- rbind(df.YEAR, c(df.YEAR$Group.1[i-1]+1, 0))
    }
    df.YEAR <- df.YEAR[order(df.YEAR$Group.1), ]
  }
}
```

```
while(df.YEAR$Group.1[length(df.YEAR$Group.1)] != 2017)
{
  df.YEAR <- rbind(df.YEAR, c(df.YEAR$Group.1[length(df.YEAR$Group.1)]+1, 0))
}
var(df.YEAR$x)/mean(df.YEAR$x)

### Figure 1
dst_march_1989 <- dst[substr(dst$DATE, 1, 4)=="1989" & substr(dst$DATE, 6, 7)=="03", ]
thresholds <- data.frame(x = seq(1:length(dst_march_1989$DST)), y = c(-50, -100, -250))
p1 <- ggplot(dst_march_1989, aes(x=seq(1:length(dst_march_1989$DST)), y=DST)) + geom_point() + geom_line() + xlab("Time (hours)") +
ylab("Dst") +
  geom_line(aes( x, y, linetype = factor(y)), thresholds) + scale_linetype_discrete(name="Magnitude", labels=c("Super storm", "Intense
storm", "Moderate storm")) +
  theme(panel.grid.major = element_blank(), panel.grid.minor = element_blank(),plot.title = element_text(hjust = 0.5),
        panel.background = element_blank(), axis.line = element_line(colour = "black"), legend.position=c(0.8, 0.2)) + ggtitle("a. Dst index in
March 1989")

quebec <- quebec[1:2000, ]
thresholds <- data.frame(x = seq(1:length(quebec$SYM.H)), y = c(50, -50, -100, -250))
p2 <- ggplot(quebec, aes(x=seq(1:length(quebec$SYM.H)), y=SYM.H)) + geom_point() + geom_line() + xlab("Time (minutes)") + ylab("SYM-
H") +
  geom_line(aes( x, y, linetype = factor(y)), thresholds) + scale_linetype_discrete(name="Magnitude", labels=c("Super storm", "Intense
storm", "Moderate storm", "50 nT")) +
  theme(panel.grid.major = element_blank(), panel.grid.minor = element_blank(),plot.title = element_text(hjust = 0.5),
        panel.background = element_blank(), axis.line = element_line(colour = "black"), legend.position=c(0.2, 0.2)) + ggtitle("b. SYM-H around
the March 1989 event")

multiplot(p1, p2, cols=2)

### Fit a Weibull distribution to inter-occurrence times
shape1 <- vector()
scale1 <- vector()
for (i in 1:26)
{
  eval(parse(text=paste0("shape1[", i,"]<-fitdist(as.numeric(na.omit(diff.times$diff", i+14,")), 'weibull')$estimate[1]")))
  eval(parse(text=paste0("scale1[", i,"]<-fitdist(as.numeric(na.omit(diff.times$diff", i+14,")), 'weibull')$estimate[2]")))
}
shape1[11]; scale1[11] # Weibull parameters corresponding to super-storms (Dst < -250nT)

### Model for shape and scale parameters (excluding inter-occurrence times below the 48h limit)
mdata <- melt(diff.times)
for (i in 1:40)
{
  eval(parse(text=paste0("mdata$threshold[mdata$variable=='diff", i, "'] <- -10*i")))
}
mdata <- mdata[!is.na(mdata$value), ]
### remove lower thresholds (independence is not clear) and fit the model
mdata <- mdata[mdata$threshold<=-150, ]

reg.weibull <- survreg(Surv(value)~threshold, data=mdata, dist="weibull")

### Figure 2 (Gnuplot v3.7)
set key bot lef rev Lef
set key spacing 2.5
set xlabel ' \vspace{-0.5cm}\LARGE inter-occurrence time $ t $ (days) \normalsize'
set ylabel ' \vspace{-3.5cm}\LARGE probability density $ f(t) $ (days$^{-1}$) \normalsize'
set data style linesp
set nogrid
set log

wei(x,a)=g/a*(x/a)**(g-1)*exp(-(x/a)**g)
g=0.68 # 0.63
b0=2.64
b1=-0.01331
expo(x)=exp(-x/mean)/mean

set yrange[1e-6:*]
set xrange[1:5000]
plot'fig2_150.txt't' \large empirical, $T=-150$ nT \normalsize' ,'col2_interoccur150.txt' u 1:2:3 notitle w e 1
```

```
T=150
a=exp(b0-b1*T)
rep wei(x,a) t' \large Weibull fit \normalsize' w l -1
mean=131.2012
rep expo(x) t' \large exponential fit \normalsize' w l 2
pause -1

plot'fig2_250.txt' t' \large empirical, $T=-250$ nT \normalsize','col2_interoccur250.txt' u 1:2:3 notitle w e 1
T=250
a=exp(b0-b1*T)
rep wei(x,a) t' \large Weibull fit \normalsize' w l -1
mean=458.421
rep expo(x) t' \large exponential fit \normalsize' w l 2

### Figure 3
par(mfrow=c(2, 3))
  plot(acf(na.omit(diff.times$diff5), plot=FALSE)[1:12],   ylim=c(-0.5, 0.5), main="a. Dst < -50 nT")
  plot(acf(na.omit(diff.times$diff10), plot=FALSE)[1:12],  ylim=c(-0.5, 0.5), main="b. Dst < -100 nT")
  plot(acf(na.omit(diff.times$diff15), plot=FALSE)[1:12],  ylim=c(-0.5, 0.5), main="c. Dst < -150 nT")
  plot(acf(na.omit(diff.times$diff20), plot=FALSE)[1:12],  ylim=c(-0.5, 0.5), main="d. Dst < -200 nT")
  plot(acf(na.omit(diff.times$diff25), plot=FALSE)[1:12],  ylim=c(-0.5, 0.5), main="e. Dst < -250 nT")
  plot(acf(na.omit(diff.times$diff30), plot=FALSE)[1:12],  ylim=c(-0.5, 0.5), main="f. Dst < -300 nT")
dev.off()

### Figure 4
par(mfrow=c(1, 2))
  plot(unique(mdata$threshold), log(shape1), xlab="Threshold", ylab="log(shape parameter)", main="a. Shape parameter")
  points(unique(mdata$threshold), rep(log(1/reg.weibull$scale), 26), type="l")
  plot(unique(mdata$threshold), log(scale1), xlab="Threshold", ylab="log(scale parameter)", main="b. Scale parameter")
  points(unique(mdata$threshold), coef(reg.weibull)[1]+coef(reg.weibull)[2]*unique(mdata$threshold), type="l")
dev.off()

### Probability of a Carrington event (Dst < -850nT) in the next decade (bootstrap)
carringtonTime <- as.numeric(difftime(Sys.Date(), as.Date("1859-09-01")))
futurTime <- as.POSIXlt(Sys.time())
futurTime$year <- futurTime$year+10
futurTime <- as.numeric(difftime(as.Date(futurTime), as.Date("1859-09-01")))
pcarr <- vector()
for (i in 1:100000)
{
  boots.df_i  <- mdata[sample(nrow(mdata), dim(mdata)[1], replace=TRUE), ]
  reg.weibull <- survreg(Surv(value)~threshold, data=boots.df_i, dist="weibull")
  shape       <- 1/reg.weibull$scale
  scale       <- exp(coef(reg.weibull)[1]+coef(reg.weibull)[2]*-850)
  pcarr[i]    <- ((1-exp(-(futurTime/scale)^(shape))) -
                 (1-exp(-(carringtonTime/scale)^(shape))))/(exp(-(carringtonTime/scale)^(shape)))
}

median(pcarr); sd(pcarr); quantile(pcarr, 0.025); quantile(pcarr, 0.975)

### Probability of a Carrington event (Dst < -850nT) in the next decade (delta method)
reg.weibull <- survreg(Surv(value)~threshold, data=mdata, dist="weibull")
carringtonTime <- as.numeric(difftime(Sys.Date(), as.Date("1859-09-01")))
futurTime <- as.POSIXlt(Sys.time())
futurTime$year <- futurTime$year+10
futurTime <- as.numeric(difftime(as.Date(futurTime), as.Date("1859-09-01")))
pr <- c(coef(reg.weibull), reg.weibull$scale)
names(pr) <- c("beta0", "beta1", "shape")
vcov.total <- as.data.frame(vcov(reg.weibull))
pcarr <- deltaMethod(pr, "((1-exp(-(futurTime/exp(beta0+beta1*-850))^(1/shape))) -
                 (1-exp(-(carringtonTime/exp(beta0+beta1*-850))^(1/shape))))/(exp(-(carringtonTime/exp(beta0+beta1*-850))^(1/shape)))",
vcov=as.matrix(vcov.total))$Estimate
se <- deltaMethod(pr, "((1-exp(-(futurTime/exp(beta0+beta1*-850))^(1/shape))) -
                 (1-exp(-(carringtonTime/exp(beta0+beta1*-850))^(1/shape))))/(exp(-(carringtonTime/exp(beta0+beta1*-850))^(1/shape)))",
vcov=as.matrix(vcov.total))$SE
pcarr; se ### probability and 95% confidence interval

### Probability of a Carrington event (Dst < -1760nT) in the next decade (bootstrap)
carringtonTime <- as.numeric(difftime(Sys.Date(), as.Date("1859-09-01")))
futurTime <- as.POSIXlt(Sys.time())
```

```
futurTime$year <- futurTime$year+10
futurTime <- as.numeric(difftime(as.Date(futurTime), as.Date("1859-09-01")))
pcarr <- vector()
for (i in 1:100000)
{
  boots.df_i  <- mdata[sample(nrow(mdata), dim(mdata)[1], replace=TRUE), ]
  reg.weibull <- survreg(Surv(value)~threshold, data=boots.df_i, dist="weibull")
  shape       <- 1/reg.weibull$scale
  scale       <- exp(coef(reg.weibull)[1]+coef(reg.weibull)[2]*-1760)
  pcarr[i]    <- ((1-exp(-(futurTime/scale)^(shape))) -
                 (1-exp(-(carringtonTime/scale)^(shape))))/(exp(-(carringtonTime/scale)^(shape)))
}

median(pcarr); sd(pcarr); quantile(pcarr, 0.025); quantile(pcarr, 0.975)

### Table 1
reg.weibull <- survreg(Surv(value)~threshold, data=mdata, dist="weibull")
# -100nT:
t1  <- 365
sh  <- 1/reg.weibull$scale
sc  <- exp(coef(reg.weibull)[1]+coef(reg.weibull)[2]*-100)
scal <- sc^(-sh)
t1p  <- sum(dWeibullCount(0:100,sh,scal,method = c("series_mat"),time=t1)*seq(0,100,1))

# -200nT:
t1  <- 365
sh  <- 1/reg.weibull$scale
sc  <- exp(coef(reg.weibull)[1]+coef(reg.weibull)[2]*-200)
scal <- sc^(-sh)
t2  <- sum(dWeibullCount(0:100,sh,scal,method = c("series_mat"),time=t1)*seq(0,100,1))

# -400nT:
t1  <- 365*10
sh  <- 1/reg.weibull$scale
sc  <- exp(coef(reg.weibull)[1]+coef(reg.weibull)[2]*-400)
scal <- sc^(-sh)
t3  <- sum(dWeibullCount(0:100,sh,scal,method = c("series_mat"),time=t1)*seq(0,100,1))

# -800nT:
t1  <- 365*1000
sh  <- 1/reg.weibull$scale
sc  <- exp(coef(reg.weibull)[1]+coef(reg.weibull)[2]*-800)
scal <- sc^(-sh)
t4  <- sum(dWeibullCount(0:100,sh,scal,method = c("series_mat"),time=t1)*seq(0,100,1))

# -1600nT:
t1  <- 365*1000000
sh  <- 1/reg.weibull$scale
sc  <- exp(coef(reg.weibull)[1]+coef(reg.weibull)[2]*-1600)
scal <- sc^(-sh)
t5  <- sum(dWeibullCount(0:100,sh,scal,method = c("series_mat"),time=t1)*seq(0,100,1))

### Table S1
fitdist(as.numeric(na.omit(diff.times$diff15)), "weibull")$aic
fitdist(as.numeric(na.omit(diff.times$diff15)), "gamma")$aic
fitdist(as.numeric(na.omit(diff.times$diff15)), "exp")$aic

fitdist(as.numeric(na.omit(diff.times$diff20)), "weibull")$aic
fitdist(as.numeric(na.omit(diff.times$diff20)), "gamma", method="mme")$aic ### MLE does not converge
fitdist(as.numeric(na.omit(diff.times$diff20)), "exp")$aic

fitdist(as.numeric(na.omit(diff.times$diff25)), "weibull")$aic
fitdist(as.numeric(na.omit(diff.times$diff25)), "gamma")$aic ### MLE does not converge
fitdist(as.numeric(na.omit(diff.times$diff25)), "exp")$aic

fitdist(as.numeric(na.omit(diff.times$diff30)), "weibull")$aic
fitdist(as.numeric(na.omit(diff.times$diff30)), "gamma", method="mme")$aic  ### MLE does not converge
fitdist(as.numeric(na.omit(diff.times$diff30)), "exp", method="mme")$aic ### MLE does not converge

### Lilliefors corrected K-S test
```

```
LcKS(na.omit(diff.times$diff15), cdf="pweibull")$p.value
LcKS(na.omit(diff.times$diff15), cdf="pgamma")$p.value
LcKS(na.omit(diff.times$diff15), cdf="pexp")$p.value

LcKS(na.omit(diff.times$diff20), cdf="pweibull")$p.value
LcKS(na.omit(diff.times$diff20), cdf="pgamma")$p.value
LcKS(na.omit(diff.times$diff20), cdf="pexp")$p.value

LcKS(na.omit(diff.times$diff25), cdf="pweibull")$p.value
LcKS(na.omit(diff.times$diff25), cdf="pgamma")$p.value
LcKS(na.omit(diff.times$diff25), cdf="pexp")$p.value

LcKS(na.omit(diff.times$diff30), cdf="pweibull")$p.value
LcKS(na.omit(diff.times$diff30), cdf="pgamma")$p.value
LcKS(na.omit(diff.times$diff30), cdf="pexp")$p.value
```

6. References

1. Fox J, Weisberg S. *An R Companion to Applied Regression*. Second. Thousand Oaks (CA): Sage; 2011. http://socserv.socsci.mcmaster.ca/jfox/Books/Companion.
2. McShane B, Adrian M, Bradlow ET, Fader PS. Count models based on Weibull interarrival times. *J Bus Econ Stat*. 2008;26(3):369-378. doi:10.1198/073500107000000278.
3. Barlow RE, Proschan F. *Statistical Theory of Reliability and Life Testing Probability Models To Begin With*. To Begin With, Silver Spring, MD; 1981. https://searchworks.stanford.edu/view/1623641. Accessed July 8, 2018.
4. Kharrat T, Boshnakov GN. Countr: Flexible Univariate Count Models Based on Renewal Processes. 2018.
